# Supplementary material for: Neurocognitive Outcomes After Extracranial Surgery and General Anesthesia in Patients with a History of Mild-to-Moderate Traumatic Brain Injury: Systemic Review and Meta-Analysis
Source: Biology (Basel). 2025 May 31;14(6):640. doi: 10.3390/biology14060640 (PMC12189922; doi:10.3390/biology14060640)
Supplement: Supplementary file 1 [file biology-14-00640-s001.zip › biology-3605215-supplementary.pdf]

SUPPLEMENTAL DATA

Contents:

Supplementary Tables

Supplementary Table S1: PubMed search strategy (21 April 2024).
Supplementary Table S2: Embase search strategy (21 April 2024).
Supplementary Table S3: Ovid Emcare search strategy (21 April 2024)
Supplementary Table S4: Global Health search strategy (21 April 2024)
Supplementary Table S5: APA PsycInfo search strategy (21 April 2024)
Supplementary Table S6: Explanations for the full-text article exclusions.
Supplementary Table S7: Leave-one-out sensitivity analyses on ICU length of stay
Supplementary Table S8: Leave-one-out sensitivity analyses on hospital length of stay

Supplementary Tables:

Supplementary Table S1: PubMed search strategy (21 April 2024).

Search	Query	Items found
#1	"brain injuries, traumatic"[MeSH Terms] OR "traumatic brain injury"[Title/Abstract] OR "TBI"[Title/Abstract] OR ("craniocerebral trauma"[MeSH Terms] OR "brain concussion"[MeSH Terms]) OR (("head"[Title/Abstract] OR "cranium"[Title/Abstract] OR "cerebrum"[Title/Abstract] OR "brain"[Title/Abstract] OR "forebrain"[Title/Abstract] OR "skull"[Title/Abstract]) AND ("injury"[Title/Abstract] OR "trauma"[Title/Abstract] OR "damag"[Title/Abstract] OR "lesion"[Title/Abstract] OR "wound"[Title/Abstract] OR "destruction"[Title/Abstract] OR "contusion"[Title/Abstract] OR "concus"[Title/Abstract] OR "fracture"[Title/Abstract]))	359,942
#2	"Dexmedetomidine"[MeSH Terms] OR "Propofol"[MeSH Terms] OR "Ketamine"[MeSH Terms] OR "Thiopental"[MeSH Terms] OR "Etomidate"[MeSH Terms] OR "Sevoflurane"[MeSH Terms] OR "Desflurane"[MeSH Terms] OR "Midazolam"[MeSH Terms] OR "Fentanyl"[MeSH Terms] OR "Rocuronium"[MeSH Terms] OR "Sufentanil"[MeSH Terms] OR "vecuronium bromide"[MeSH Terms] OR "Morphine"[MeSH Terms] OR "Dexmedetomidine"[Title/Abstract] OR "Propofol"[Title/Abstract] OR "Ketamine"[Title/Abstract] OR "Thiopental"[Title/Abstract] OR "Etomidate"[Title/Abstract] OR "Sevoflurane"[Title/Abstract] OR "Isoflurane"[Title/Abstract] OR "Desflurane"[Title/Abstract] OR "Midazolam"[Title/Abstract] OR "Ketofol"[Title/Abstract] OR "Fentanyl"[Title/Abstract] OR "Rocuronium"[Title/Abstract] OR "Sufentanil"[Title/Abstract] OR "Vecuronium"[Title/Abstract] OR "Morphine"[Title/Abstract]	174,245
#3	"extracranial Surgery"[Title/Abstract] OR "orthopedic surgery"[Title/Abstract] OR "orthopedic surgeries"[Title/Abstract] OR "limb injury"[Title/Abstract] OR "limb injuries"[Title/Abstract] OR "knee injury"[Title/Abstract] OR "knee injuries"[Title/Abstract] OR "knee surgery"[Title/Abstract] OR "hip injury"[Title/Abstract]	118,171

	OR "hip injuries"[Title/Abstract] OR "hip fractures"[Title/Abstract] OR "maxillofacial surgery"[Title/Abstract] OR "facial injury"[Title/Abstract] OR "facial injuries"[Title/Abstract] OR "facial fractures"[Title/Abstract] OR "dental injuries"[Title/Abstract] OR "tooth injury"[Title/Abstract] OR "Colonoscopy"[Title/Abstract] OR "thoracic surgery"[Title/Abstract] OR "rib fractures"[Title/Abstract] OR "abdominal trauma"[Title/Abstract] OR "abdominal injuries"[Title/Abstract] OR "abdominal injury"[Title/Abstract] OR "crush injuries"[Title/Abstract] OR "crush injury"[Title/Abstract]	
#4	(#2 OR #3)	289,791
#5	(#1 AND #4)	10,247
#6	"cohort studies"[MeSH Terms] OR "case-control studies"[MeSH Terms] OR "comparative study"[Publication Type] OR "risk factors"[MeSH Terms] OR "cohort"[Text Word] OR "compared"[Text Word] OR "groups"[Text Word] OR "case control"[Text Word] OR "multivariate"[Text Word]	9,879,849
#7	(#5 AND #6)	4,728

Supplementary Table S2: Embase search strategy (21 April 2024).

Search	Query	Items found
#1	exp "brain injuries, traumatic"/ or "traumatic brain injury".tw. or TBI.tw. or (exp "craniocerebral trauma"/ or exp "brain concussion"/) or ((head or cranium or cerebrum or brain or forebrain or skull) and (injury or trauma or damag or lesion or wound or destruction or contusion or concus or fracture)).tw.	550027
#2	exp Dexmedetomidine/ or exp Propofol/ or exp Ketamine/ or exp Thiopental/ or exp Etomidate/ or exp Sevoflurane/ or exp Desflurane/ or exp Midazolam/ or exp Fentanyl/ or exp Rocuronium/ or exp Sufentanil/ or exp "vecuronium bromide"/ or exp Morphine/ or Dexmedetomidine.tw. or Propofol.tw. or Ketamine.tw. or Thiopental.tw. or Etomidate.tw. or Sevoflurane.tw. or Isoflurane.tw. or Desflurane.tw. or Midazolam.tw. or Ketofol.tw. or Fentanyl.tw. or Rocuronium.tw. or Sufentanil.tw. or Vecuronium.tw. or Morphine.tw.	370880

#3	("extracranial Surgery" or "orthopedic surgery" or "orthopedic surgeries" or "limb injury" or "limb injuries" or "knee injury" or "knee injuries" or "knee surgery" or "hip injury" or "hip injuries" or "hip fractures" or "maxillofacial surgery" or "facial injury" or "facial injuries" or "facial fractures" or "dental injuries" or "tooth injury" or Colonoscopy or "thoracic surgery" or "rib fractures" or "abdominal trauma" or "abdominal injuries" or "abdominal injury" or "crush injuries" or "crush injury =").tw.	178396
#4	2 or 3	543186
#5	1 and 4	19917
#6	exp 'clinical article'/ or exp 'controlled study'/ or exp 'major clinical study'/ or exp 'prospective study'/ or exp 'cohort analysis'/ or 'cohort'.ti,ab. or 'compared'.ti,ab. or 'groups'.ti,ab. or 'case control'.ti,ab. or 'multivariate'.ti,ab.	18873448
#7	5 and 6	11942

Supplementary Table S3: Ovid Emcare search strategy (21 April 2024).

Search	Query	Items found
#1	exp "brain injuries, traumatic"/ or "traumatic brain injury".tw. or TBI.tw. or (exp "craniocerebral trauma"/ or exp "brain concussion"/) or ((head or cranium or cerebrum or brain or forebrain or skull) and (injury or trauma or damag or lesion or wound or destruction or contusion or concus or fracture)).tw.	114780
#2	exp Dexmedetomidine/ or exp Propofol/ or exp Ketamine/ or exp Thiopental/ or exp Etomidate/ or exp Sevoflurane/ or exp Desflurane/ or exp Midazolam/ or exp Fentanyl/ or exp Rocuronium/ or exp Sufentanil/ or exp "vecuronium bromide"/ or exp Morphine/ or Dexmedetomidine.tw. or Propofol.tw. or Ketamine.tw. or Thiopental.tw. or Etomidate.tw. or Sevoflurane.tw. or Isoflurane.tw. or Desflurane.tw. or Midazolam.tw. or Ketofol.tw. or Fentanyl.tw. or Rocuronium.tw. or Sufentanil.tw. or Vecuronium.tw. or Morphine.tw.	92744
#3	("extracranial Surgery" or "orthopedic surgery" or "orthopedic surgeries" or "limb injury" or "limb injuries" or "knee injury" or	48434

	"knee injuries" or "knee surgery" or "hip injury" or "hip injuries" or "hip fractures" or "maxillofacial surgery" or "facial injury" or "facial injuries" or "facial fractures" or "dental injuries" or "tooth injury" or Colonoscopy or "thoracic surgery" or "rib fractures" or "abdominal trauma" or "abdominal injuries" or "abdominal injury" or "crush injuries" or "crush injury =").tw.	
#4	2 or 3	139313
#5	1 and 4	5100
#6	exp 'clinical article'/ or exp 'controlled study'/ or exp 'major clinical study'/ or exp 'prospective study'/ or exp 'cohort analysis'/ or 'cohort'.ti,ab. or 'compared'.ti,ab. or 'groups'.ti,ab. or 'case control'.ti,ab. or 'multivariate'.ti,ab.	3465306
#7	5 and 6	2640

Supplementary Table S4: Global Health search strategy (21 April 2024).

Search	Query	Items found
#1	exp "brain injuries, traumatic"/ or "traumatic brain injury".tw. or TBI.tw. or (exp "craniocerebral trauma"/ or exp "brain concussion"/) or ((head or cranium or cerebrum or brain or forebrain or skull) and (injury or trauma or damag or lesion or wound or destruction or contusion or concus or fracture)).tw.	15828
#2	exp Dexmedetomidine/ or exp Propofol/ or exp Ketamine/ or exp Thiopental/ or exp Etomidate/ or exp Sevoflurane/ or exp Desflurane/ or exp Midazolam/ or exp Fentanyl/ or exp Rocuronium/ or exp Sufentanil/ or exp "vecuronium bromide"/ or exp Morphine/ or Dexmedetomidine.tw. or Propofol.tw. or Ketamine.tw. or Thiopental.tw. or Etomidate.tw. or Sevoflurane.tw. or Isoflurane.tw. or Desflurane.tw. or Midazolam.tw. or Ketofol.tw. or Fentanyl.tw. or Rocuronium.tw. or Sufentanil.tw. or Vecuronium.tw. or Morphine.tw.	6979
#3	("extracranial Surgery" or "orthopedic surgery" or "orthopedic surgeries" or "limb injury" or "limb injuries" or "knee injury" or "knee injuries" or "knee surgery" or "hip injury" or "hip injuries" or "hip fractures" or "maxillofacial surgery" or "facial injury" or "facial	9101

	injuries" or "facial fractures" or "dental injuries" or "tooth injury" or Colonoscopy or "thoracic surgery" or "rib fractures" or "abdominal trauma" or "abdominal injuries" or "abdominal injury" or "crush injuries" or "crush injury =").tw.	
#4	2 or 3	15998
#5	1 and 4	431

Supplementary Table S5: APA PsycInfo search strategy (21 April 2024).

Search	Query	Items found
#1	exp "brain injuries, traumatic"/ or "traumatic brain injury".tw. or TBI.tw. or (exp "craniocerebral trauma"/ or exp "brain concussion"/) or ((head or cranium or cerebrum or brain or forebrain or skull) and (injury or trauma or damag or lesion or wound or destruction or contusion or concus or fracture)).tw.	59778
#2	exp Dexmedetomidine/ or exp Propofol/ or exp Ketamine/ or exp Thiopental/ or exp Etomidate/ or exp Sevoflurane/ or exp Desflurane/ or exp Midazolam/ or exp Fentanyl/ or exp Rocuronium/ or exp Sufentanil/ or exp "vecuronium bromide"/ or exp Morphine/ or Dexmedetomidine.tw. or Propofol.tw. or Ketamine.tw. or Thiopental.tw. or Etomidate.tw. or Sevoflurane.tw. or Isoflurane.tw. or Desflurane.tw. or Midazolam.tw. or Ketofol.tw. or Fentanyl.tw. or Rocuronium.tw. or Sufentanil.tw. or Vecuronium.tw. or Morphine.tw.	20653
#3	("extracranial Surgery" or "orthopedic surgery" or "orthopedic surgeries" or "limb injury" or "limb injuries" or "knee injury" or "knee injuries" or "knee surgery" or "hip injury" or "hip injuries" or "hip fractures" or "maxillofacial surgery" or "facial injury" or "facial injuries" or "facial fractures" or "dental injuries" or "tooth injury" or Colonoscopy or "thoracic surgery" or "rib fractures" or "abdominal trauma" or "abdominal injuries" or "abdominal injury" or "crush injuries" or "crush injury =").tw.	2346
#4	2 or 3	22936
#5	1 and 4	494

Supplementary Table S6. Explanations for the full-text article exclusions.

SL/NO	Title	Reasons
1	Dynamic change in cortisol levels associated with severity, progression, and survival of patients with traumatic brain injury.	Unrelated population
2	Sedative exposure and cognitive outcomes following pediatric critical care.	Unrelated study design
3	Intraoperative dexmedetomidine versus midazolam in patients undergoing peripheral surgery with mild traumatic brain injuries: A retrospective cohort analysis.	Unrelated population
4	Acute secondary adrenal insufficiency after traumatic brain injury: a prospective study.	Unrelated population
5	Surgical stabilization versus nonoperative treatment for flail and non-flail rib fracture patterns in patients with traumatic brain injury.	Unrelated outcome
6	Clinical Study of Dexmedetomidine in Combination with Butorphanol for the Treatment of Traumatic Brain Injury (TBI).	Unrelated population
7	Glial fibrillary acidic protein is highly correlated with brain injury.	Unrelated population
8	Complex pediatric orbital fractures combined with traumatic brain injury: treatment and follow-up.	Unrelated control
9	Cerebral autoregulation in pediatric traumatic brain injury.	Unrelated control
10	Early appropriate care: definitive stabilization of femoral fractures within 24 hours of injury is safe in most patients with multiple injuries.	Unrelated outcome
11	Timing of surgery after multisystem injury with traumatic brain injury: effect on neuropsychological and functional outcome.	Unrelated control
12	Morbidity and mortality in elderly trauma patients.	Unrelated outcome
13	Assessment of the relationship between timing of fixation of the fracture and secondary brain injury in patients with multiple trauma.	Unrelated population
14	Timing of Femur Fracture Fixation: Effect on Outcome in Patients with Thoracic and Head Injuries	Unrelated population
15	Lower extremity fracture fixation in head-injured patients.	Unrelated outcome
16	Early fracture fixation may be "just fine" after head injury: no difference in central nervous system outcomes.	Unrelated outcome

17	Operative management of lower extremity fractures in patients with head injuries.	Unrelated control
18	Alterations in the systemic inflammatory response after early total care and damage control procedures for femoral shaft fracture in severely injured patients.	Unrelated population
19	Risk factors and impact on long-term outcomes of early systemic insults after TBI. A CENTER-TBI study	Unrelated population
20	Adult traumatic brain injury: Identifying predictors of posttraumatic amnesia in the acute care setting.	Unrelated outcome
21	Effect of Dexmedetomidine on Oxidative Stress Response and Expression of Intracellular Adhesion Factor-1 (ICAM-1) and S100B in Patients with Traumatic Brain Injury	Unrelated population
22	Timely surgical fixation confers beneficial outcomes in patients' concomitant flail chest with mild-to-moderate traumatic brain injury: a trauma quality improvement project analysis - a cohort study	Unrelated population
23	Exposure to Sedation and Analgesia Medications: Short-term Cognitive Outcomes in Pediatric Critical Care Survivors with Acquired Brain Injury.	Unrelated population
24	Outcome related to general anaesthesia versus regional anaesthesia for lower extremity surgery in patients with mild traumatic brain injury (Gara trial)	Unrelated study design

Supplementary Table S7: Leave-one-out sensitivity analyses on ICU length of stay

	**MD [95%CI]**	**Between-study heterogeneity**
**Overall**	1.94 [-1.25, 5.13]	p =0.137; I^2=81.40%
**Omitted study**		
Shapiro et al. 2001	1.79 [-3.92, 7.50]	p =0.54; I^2=91.84%
Prins et al. 2021	3.43 [1.00, 5.85]	p =0.01; I^2=80.30%
Roberts et al. 2023	0.56 [-2.68, 3.80]	p =0.73; I^2=67.13%

Supplementary Table S8: Leave-one-out sensitivity analyses on hospital length of stay

	**MD [95%CI]**	**Between-study heterogeneity**
**Overall**	0.99 [-1.59, 3.57]	p =0.45; I^2=23.1%
**Omitted study**		
McKee et al. 1997	1.32 [-1.90, 4.54]	p =0.42; I^2=42.94%
Richards et al. 2011	0.36 [-2.75, 3.48]	p =0.82; I^2=37.59%
Shapiro et al. 2001	0.05 [-3.11, 3.21]	p =0.97; I^2=20.68%
Prins et al. 2021	2.14 [-0.38, 4.65]	p =0.10; I^2=0.00%
